# Successes and challenges of best practice alerts to identify and engage individuals living with hepatitis C virus

**DOI:** 10.3389/fpubh.2024.1281079

**Published:** 2024-05-20

**Authors:** Saniya Tandon, Roselyn Castaneda, Nadia Tarasco, Janie Percival, Roberto Nieto Linares, Glen Geiger, Curtis L. Cooper

**Affiliations:** ^1^Department of Infectious Diseases, Ottawa Hospital Research Institute, Ottawa, ON, Canada; ^2^School of Epidemiology and Public Health, University of Ottawa, Ottawa, ON, Canada; ^3^The Ottawa Hospital, Ottawa, ON, Canada

**Keywords:** hepatitis C virus (HCV), successes and challenges, linkage to care (LTC), best practice alert (BPA), electronic medical record (EMR) systems

## Abstract

**Introduction:**

Many individuals living with hepatitis C virus (HCV) are unaware of their diagnosis and/or have not been linked to programs providing HCV care. The use of electronic medical record (EMR) systems may assist with HCV infection identification and linkage to care.

**Methods:**

In October 2021, we implemented HCV serology-focused best practice alerts (BPAs) at The Ottawa Hospital (TOH) via our EMR (EPIC). Our BPAs were programmed to identify previously tested HCV seropositive individuals. Physicians were prompted to conduct HCV RNA testing and submit consultation requests to the TOH Viral Hepatitis Program. We evaluated data post-BPA implementation to assess the design and related outcomes.

**Results:**

From 1 September 2022 to 15 December 2022, a total of 2,029 BPAs were triggered for 139 individuals. As a consequence of the BPA prompts, nine HCV seropositive and nine HCV RNA-positive individuals were linked to care. The proportion of total consultations coming from TOH physicians increased post-BPA implementation. The BPA alerts were frequently declined, and physician engagement with our BPAs varied across specialty groups. Programming issues led to unnecessary BPA prompts (e.g., no hard stop to the prompts even though the individual was treated and cured and individuals linked to care without first undergoing HCV RNA testing). A fixed 6-month lookback period for test results limited our ability to identify many individuals.

**Conclusion:**

An EMR-based BPA can assist with the identification and engagement of HCV-infected individuals in care. However, challenges including issues with programming, time commitment toward BPA configuration, productive communication between healthcare providers and the programming team, and physician responsiveness to the BPAs require attention to optimize the impact of BPAs.

## Introduction

Hepatitis C virus (HCV) is a preventable and curable infection of the liver (1). If untreated, HCV can result in liver cirrhosis, liver failure, and hepatocellular carcinoma (1). Globally, 58 million people are chronically infected, and an estimated 1.5 million new infections occur annually (2). In Canada, HCV infection remains a public health concern of utmost priority and is associated with a decreased quality of life and increased healthcare costs (3, 4). In 2019, 11,441 new cases were reported in Canada (1). These cases were primarily found in persons who inject drugs (PWID) and immigrants (5–9). The prevalence of PWID in Canada increased by ~32% between 2011 and 2016 (5). The COVID-19 pandemic negatively impacted people who use drugs (10). There was an increase in the frequency of drug use as well as disruption in supplies, resulting in detrimental use patterns (10). HCV infection is often asymptomatic in those without advanced liver disease (9, 11). Therefore, individuals must be screened and linked to care, including direct-acting antiviral (DAA) treatment, before developing complications.

Electronic medical record (EMR) systems are currently pervasive and can be used in disease screening and care linkage efforts (12). A systematic review of the literature identified an increase in screening rates for people living with HCV, regardless of the type of strategies implemented [i.e., best practice alerts (BPAs), Plan–Do–Study–Action cycles, or machine learning algorithms to check for eligible people based on various risk factors] through the EMR systems (13). Most of the included studies used electronic clinical decision support (eCDS)/BPAs, which resulted in an increase in the average screening rate by ~45% (13). Another review by Haridy et al., which included 29 studies evaluating EMR alerts targeted toward the birth cohort or at-risk screening, highlighted increased screening rates (14). Through a literature search of articles published between 2020 and 2023, our group identified six additional studies that used BPAs to identify people living with HCV (15–20). While studies consistently reported an increase in screening rates, only a few examined whether the use of BPAs via an EMR system assisted in linking HCV patients to care (16, 17). All the included studies in these reviews were conducted in the United States. Additionally, the majority of the studies implemented specific birth cohort population screening (i.e., baby boomers) strategies impacting their generalizability to other settings.

There are multiple gaps along the HCV cascade of care. In an Ontario, Canadian-based evaluation, 76% of HCV seropositive individuals underwent HCV RNA testing (21). Out of those who underwent HCV RNA testing, 61% tested positive (21). Many of those who tested positive were not linked to care, resulting in reduced treatment initiations and missed opportunities to cure HCV (22, 23). Although not formally evaluated, our own experience at The Ottawa Hospital (TOH) was that both in- and out-patients treated for other medical conditions were often tested for HCV infection but not linked to care with The Ottawa Hospital Viral Hepatitis Program (TOHVHP) when these results were found positive. Additionally, multiple HCV serology tests were performed without follow-up HCV RNA testing. Evidence suggests that EMR systems can be a powerful tool for identifying people living with HCV and facilitating their progression along the HCV care continuum. We describe the development, implementation, and outcomes related to the use of EMR HCV-specific BPAs in a Canadian, public health-funded, tertiary care hospital setting. The objectives of this evaluation included the following: (1) to assess the design and implementation of HCV serology-focused BPAs; (2) to examine if these BPAs led to appropriate testing and linkage to the care of HCV-infected individuals; and (3) to highlight the successes and challenges of our current EMR BPA configuration.

## Methods

### Electronic medical record BPA development and implementation

**Figure 1:**
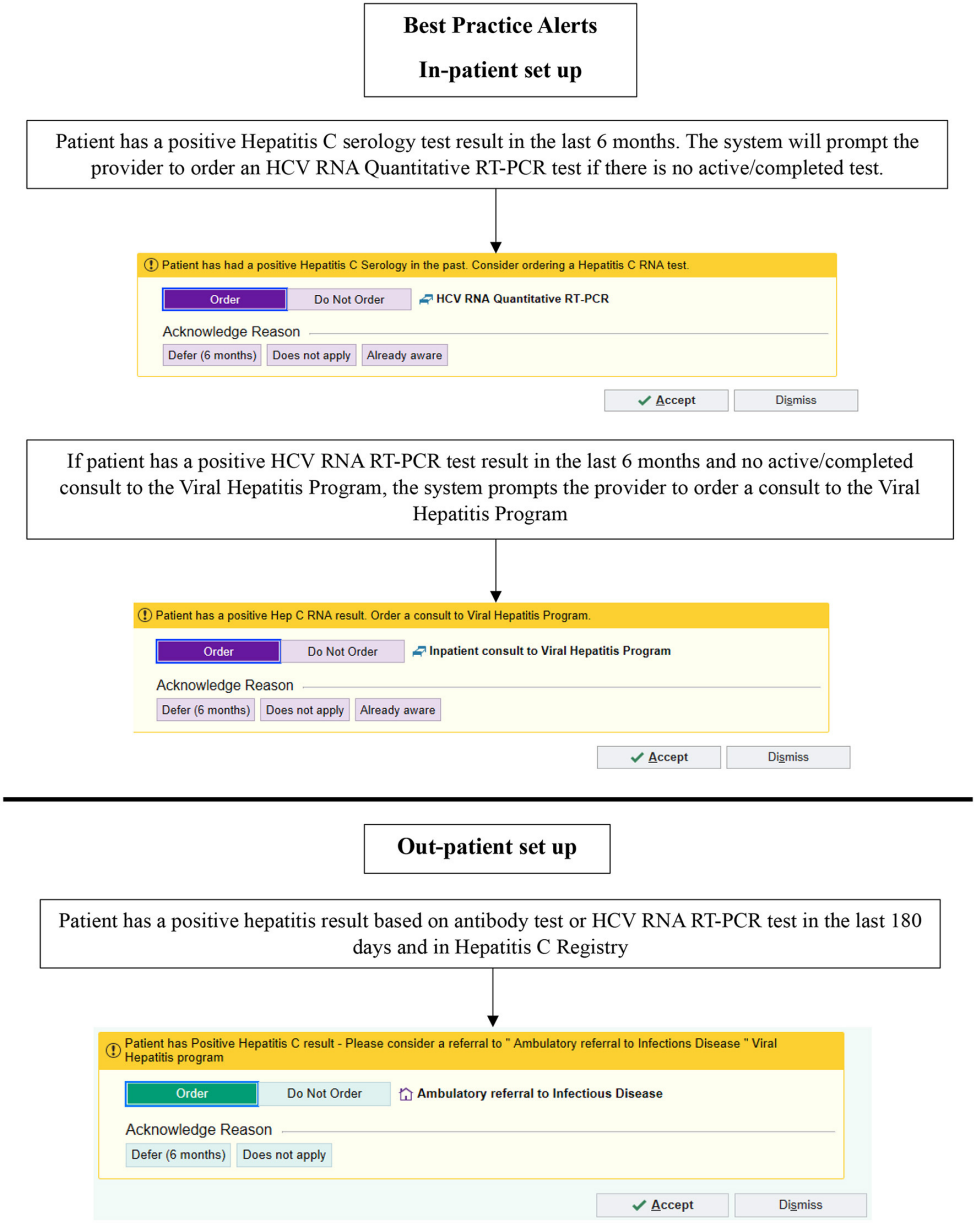
Best practice alerts implemented using EPIC in September 2021. HCV, hepatitis C virus; RNA, ribonucleic acid; PCR, polymerase chain reaction. © 2024 Epic Systems Corporation.

From October 2019, the TOHVHP group began developing a set of EPIC EMR BPAs for in- and out-patient care. The objectives of implementing these BPAs included the following: (1) to increase identification of patients living with HCV; (2) to ensure appropriate HCV RNA testing to confirm chronic infection; (3) to reduce repetitive HCV serology testing, and (4) to ensure that patients in need of treatment were linked to TOHVHP. The BPAs for appropriate HCV screening were launched in September 2021. We aimed to develop these BPAs to identify HCV seropositive individuals and encourage physicians to carry out HCV RNA testing (Figure 1). If the patient was found to be HCV RNA-positive, then the BPA would advise the physician to request a TOHVHP consultation. To enable an order/consultation to be placed, the BPAs were configured to trigger whenever a patient's chart was opened by a physician.

In the out-patient setting, if the patient had a positive HCV serology or HCV RNA test in the preceding 6 months, an in-basket message was sent to the healthcare provider recommending a TOHVHP consultation for HCV treatment. Providers/users had the following options if a referral was not ordered: “Defer (6 months)” or “Does not apply.” In the in-patient setting, if the search algorithm detected a positive HCV serology test result within the preceding 6 months, then the BPA prompted the physician to carry out an HCV RNA test. If the search algorithm detected a positive HCV RNA test result in the preceding 6 months and there was no active/completed TOHVHP consultation, the system prompted a BPA suggesting TOHVHP consultation submission. Once a test/consultation to TOHVHP was ordered, the BPAs ceased triggering. Upon the presentation of the BPA on the screen, providers/users could select from the following options if a test/consultation to TOHVHP was not ordered: “Defer (6 months),” “Does not apply,” and “Already aware”.

The providers/users also had the option to dismiss the BPA for a current encounter. If a BPA was dismissed, it would not launch until the next time an encounter was opened for the patient. If an out-patient was previously treated or currently engaged in treatment, the BPAs would continue to be triggered based on a positive test result within the 6-month time frame. If a consult to TOHVHP was submitted while an individual was an in-patient, an out-patient BPA would still be triggered once they were discharged. An abnormal test result (positive test result for serology or HCV RNA), as indicated on a patient's file, triggered the BPA. Healthcare providers were formally notified when the BPAs were launched, but no formal training or education to respond to these BPAs was provided.

### BPA evaluation

An EPIC workbench report (“My reports”) was generated as part of our post-implementation evaluation of our BPAs. This report captured patient- and user-level information, such as the number of triggers and actions taken during a 4-month period after implementation (1 September 2022 to 15 December 2022). The report allowed for the following information to be extracted: number of BPA triggers, actions taken when the BPA was triggered, reasons for declining the BPA trigger, and identifying the type of healthcare provider receiving these BPAs. An EPIC medical record review was conducted for each patient triggering a BPA. Details were collected pertaining to demographics (age and gender), serology and HCV RNA tests undergone by patients, patient linkage to care, number of patients currently being treated at the TOHVHP, and patients already treated and deemed cured. All data points were extracted on Microsoft Excel and analyzed descriptively using SAS version 9.4 to provide cumulative frequencies and percentages for categorical data and median (interquartile range—IQR) for continuous data.

## Results

### Physician-specific BPA outcomes

**Figure 2:**
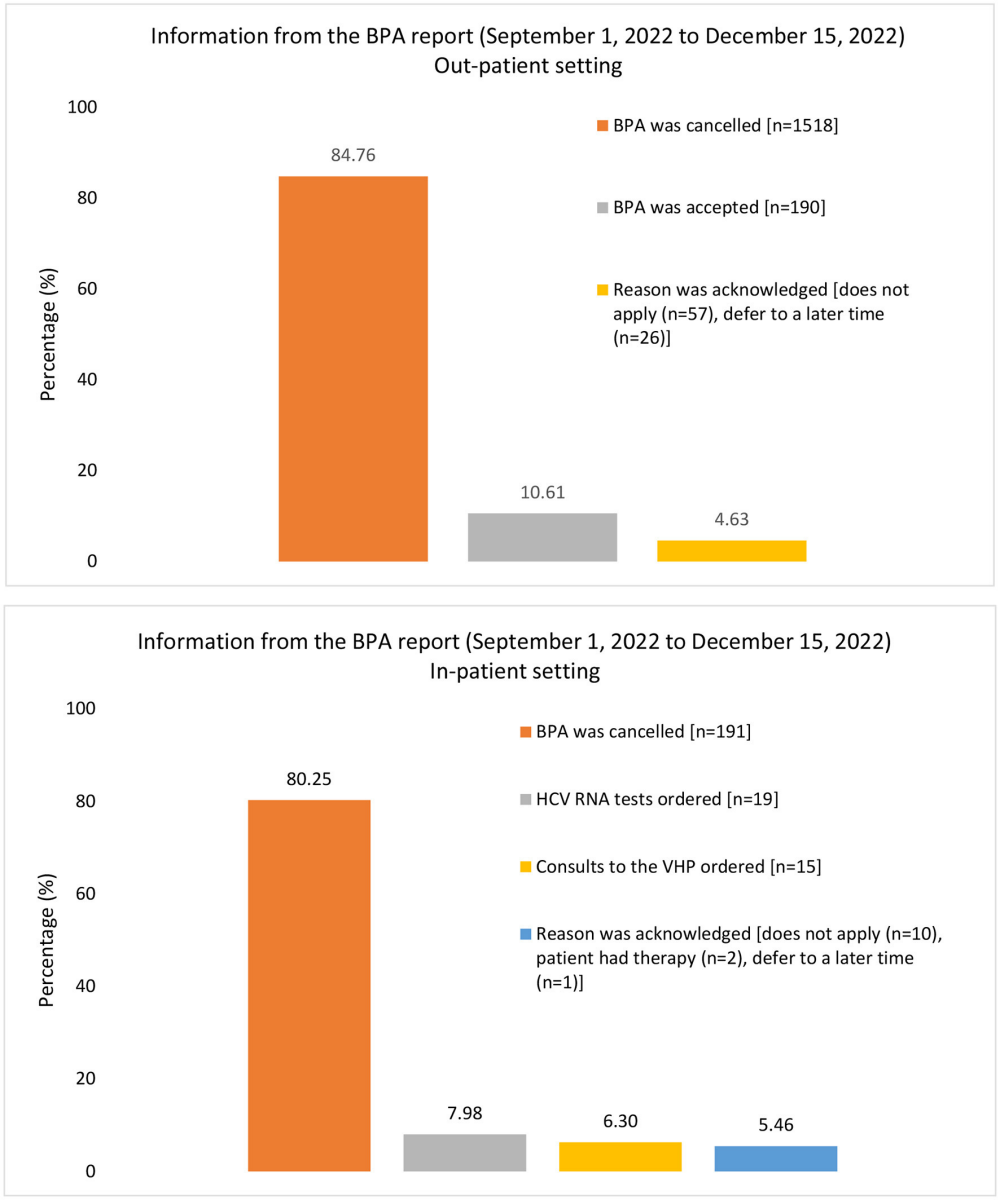
Information captured from the report for BPA triggers from 1 September 2022, to 15 December 2022. There were a total of 1,791 outpatient BPA triggers and 238 inpatient triggers, which were used as the denominator for each graph respectively. The proportion of BPAs acted upon or canceled are presented as percentages. The information recorded in the BPA report differs between inpatient and outpatient settings, leading to a difference in the column presentation for this figure. BPA, best practice alert; HCV, hepatitis C virus; RNA, ribonucleic acid; VHP, viral hepatitis program.

**Figure 3:**
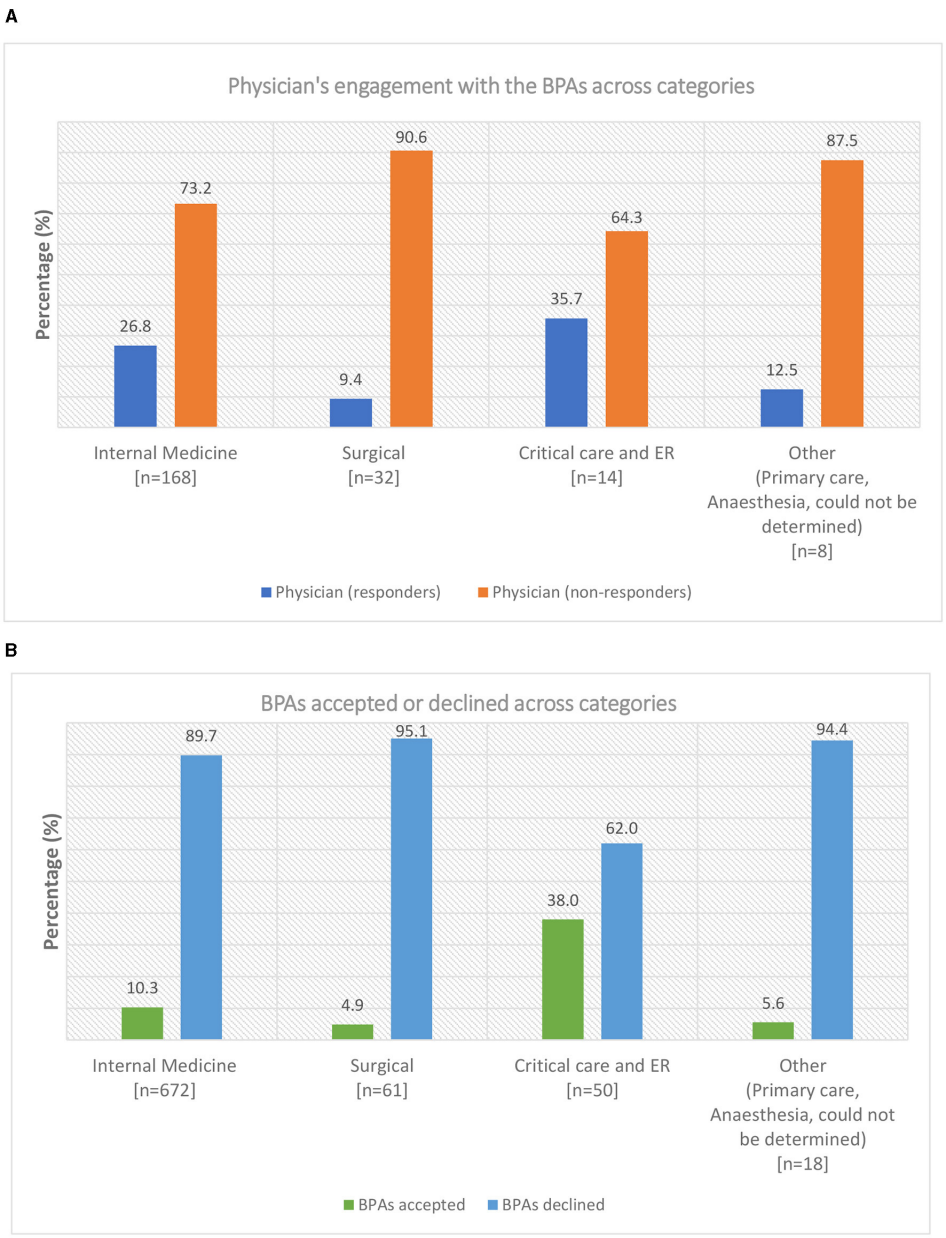
**(A)** Physician's engagement with the best practice alerts (BPAs) across various categories at The Ottawa Hospital. The specialties were grouped into four categories: internal medicine, surgical, critical care and emergency room (ER), and others (primary care, anesthesia, could not be determined). The total number of physicians (*n*) is presented under each category. A physician taking a BPA action for one or multiple patients was classified as a responder. A physician canceling all BPAs encountered was classified as a non-responder. The proportion of physicians who responded or did not respond to the BPAs is presented as a percentage. **(B)** Best practice alerts (BPAs) acted upon or declined across categories at The Ottawa Hospital. The total number of BPAs triggered (*n*) is presented under each category. The proportion of BPAs acted upon or declined across the categories is presented as a percentage. Data do not include BPAs triggered for non-physicians.

During the evaluation period between 1 September and 15 December 2022, 2,029 BPA messages were triggered (1,791 from outpatient clinics and 238 from the inpatient setting; Figure 2). The 238 in-patient BPAs were triggered for 60 individual physicians and no other healthcare providers. In response to these in-patient BPAs, 19 orders were placed for HCV RNA testing and 15 orders for TOHVHP consultation were submitted. In addition, the reason for acknowledgment was recorded 13 times [patient aware (*n* = 2)/defer to a later time (*n* = 1)/does not apply (*n* = 10)], and the BPAs were canceled 191 times. As a restriction was not placed at the time of the build for out-patient BPAs, the triggers were prompted appropriately for 222 individual physicians an inappropriately for 415 individual non-physicians. The BPAs were accepted 190 times, canceled 1,518 times, and acknowledged 83 times [defer to a later time (*n* = 26)/does not apply (*n* = 57)]. BPAs were triggered for physicians across various specialties. We grouped individual physicians by specialty into four categories: internal medicine (*n* = 168), surgical (*n* = 32), critical care and emergency room (ER; *n* = 14), and other (anesthesia, primary care, could not be determined; *n* = 8; Figure 3A). Physician engagement with the BPAs was highest for critical care and ER and lowest for surgical subspecialties (*p* < 0.05). The absolute number of BPA triggers varied across these specialty categories: internal medicine (*n* = 672), surgical (*n* = 61), critical care and ER (*n* = 50), and other (*n* = 18; Figure 3B). The proportion of BPAs declined varied by specialty grouping: surgical (95.1%), internal medicine (89.7%), critical care and ER (62.0%).

### Patient-specific BPA outcomes

As mentioned above, in-patient and out-patient BPAs were triggered for a total of 139 individual patients (Table 1). Out-patient BPAs were triggered for 125 (89.9%) patients, in-patient BPAs were triggered for six (4.3%) patients, and both (in-patient and out-patient) BPAs were triggered for eight (5.8%) patients. Two-thirds of patients (68%) were men. The median age was 57 years (IQR: 18.0). Of the 139 patients, 34 (24.5%) had already received antiviral treatment and achieved sustained virological response (SVR), one (0.7%) had received antiviral treatment and was due for SVR bloodwork, 18 (12.9%) had spontaneously cleared the infection, 12 (8.6%) were actively receiving treatment, 12 (8.6%) were HCV RNA-positive (six of which were known to the TOHVHP), and 15 (10.8%) were HCV seropositive with no HCV RNA test result (see Table 1 for the entire list of patient profiles). In several instances, providers ordered both HCV serology and HCV RNA testing together for a specific patient. If the HCV serology test result was negative, the HCV RNA test result would be “Not performed” due to no serological evidence. The test result “Not performed” was identified as abnormal data by the BPA alert system, leading to an erroneous BPA trigger. Additionally, a baby born to a previously HCV-treated mother had a positive HCV serology test at 5 months.

**Table 1:** Summary of patient profiles (in- and out-patients) for which BPAs were triggered from 1 September 2022 to 15 December 2022.

**Characteristics**	**Number of observations [*n* (%)]**
	**Total number of patients (*****n*** = **139)**
Patients for which outpatient BPAs were triggered	125 (89.9)
Patients for which inpatient BPAs were triggered	6 (4.3)
Patients for which both (outpatient and inpatient) BPAs were triggered	8 (5.8)
**Demographics**	
Men	95 (68.4)
Women	44 (31.6)
Age, years [median (IQR)]^*^	57 (18.0)
**Patient profiles**	
Treated and achieved SVR	34 (24.5)
Treated but due for SVR bloodwork	1 (0.7)
Spontaneous clearance	18 (12.9)
Currently on HCV antiviral treatment	12 (8.6)
Positive HCV RNA	10 (7.2)
Positive HCV RNA (no show to appointment/cannot be reached/refused treatment)	2 (1.4)
Positive serology but no HCV RNA testing	15 (10.8)
Positive serology and negative HCV RNA result	32 (23.0)
Erroneous trigger^**^	11 (7.9)
Inconclusive serology and negative HCV RNA test	1 (0.7)
Inconclusive serology and needs further testing	1 (0.7)
False-positive results (based on multiple serology tests)	1 (0.7)
Serological testing not performed due to insufficient volume	1 (0.7)

^*^A baby born to an HCV-treated mother had a positive HCV serology test at 5 months.

^**^If an HCV RNA test was not performed due to no serological evidence, the result “not performed” was identified as abnormal data by the system, which led to erroneous BPA triggers.

BPAs, best practice alerts; IQR, interquartile range; HCV, hepatitis C virus; RNA, ribonucleic acid; SVR, sustained virological response.

We conducted a chart review of inpatients and outpatients who triggered a BPA based on prior positive/inconclusive HCV serology testing results (*n* = 74; Table 2). We excluded those who had received HCV treatment and were deemed cured (*n* = 34), those who received treatment and were due for SVR bloodwork (*n* = 1), those who had spontaneously cleared prior infection (*n* = 18), those who had an erroneous BPA trigger (*n* = 11), and if a test was not performed due to an insufficient blood volume to assess specimen (*n* = 1). Out of the 74 patients with a positive or inconclusive HCV serology result, 25 (33.8%) had a positive HCV RNA test result, 35 (47.3%) had a negative HCV RNA test result, and 14 (18.9%) did not have an HCV RNA test ordered. A TOHVHP consultation was ordered as a consequence of BPA messaging for 18 patients, of which nine were HCV seropositive and nine were HCV RNA-positive. Consultations to TOHVHP were not submitted despite BPA messaging for five HCV RNA-positive patients during the analyzed time period. Furthermore, 10 patients for which a BPA was triggered were referred to the TOHVHP outside of the BPA messaging. In these cases, consultations to TOHVHP were submitted by external referral (i.e., referrals from community health centers).

**Table 2:** Overview of patients (inpatient and outpatients) with positive/inconclusive serology results triggering BPAs that were followed by appropriate confirmatory testing and linkage to care through an extensive chart review.

	**Total *n* (%)**
**Serology test result (*****N*** = **74)**	
Positive	71 (94.6)
Inconclusive	3 (5.4)
**HCV RNA test result (*****N*** = **74)**	
Positive	25 (33.8)
Negative	35 (47.3)
HCV RNA testing not conducted	14 (18.9)
**Consult to TOHVHP (*****N*** = **23)**	
Consult placed after a positive/inconclusive serology test result	9 (39.1)
Consult placed after a positive HCV RNA test result	9 (39.1)
Consult not placed despite positive HCV RNA test result	5 (21.7)

HCV, Hepatitis C virus; RNA, Ribonucleic acid; TOHVHP, The Ottawa Hospital Viral Hepatitis Program.

Individuals who received antiviral treatment and were deemed cured (n = 34) were spontaneously cleared of infection (n = 18), received treatment and were due for SVR bloodwork (n = 1), had an erroneous BPA trigger (n = 11), and if a test was not performed due to insufficient sample (n = 1) were excluded from this analysis.

In the 3-month period before BPA implementation (June to August 2021), 22.8% (13 of 57) of HCV referrals came from TOH physicians. In the 3 months immediately post-launch (September to December 2021), the number of referrals increased to 49.0% (24 of 49). This increased proportion was retained 1 year after the launch [37.0% (20 of 54), September to December 2022].

## Discussion

This evaluation highlights the successes and challenges of implementing HCV-specific BPAs via an EMR system in a publicly funded, Canadian tertiary care hospital setting. Using an EMR-based prompt for HCV seropositive individuals notified and facilitated physicians to order appropriate tests and consultations to the TOHVHP. By reviewing our HCV BPA data, we were able to distinguish between previously cured individuals, those currently on HCV antiviral treatment, and those requiring linkage to HCV care. Our data suggest that the absolute number and proportion of consultations directed to the TOHVHP from TOH physicians increased as a consequence of our BPA initiative.

Electronic medical record-based BPAs provide a powerful opportunity to identify, engage, and retain HCV-infected individuals in care. However, these BPAs were frequently ignored/canceled by our physicians. Our analysis suggests that the uptake of BPA recommendations varied by the specialty group. This knowledge is relevant in future efforts to increase and maintain physician engagement in EMR-based BPA initiatives. In-patient BPAs only prompted physicians who could act on the information. Due to a programming issue, out-patient BPAs were triggered for both physicians and non-physicians. The latter group of healthcare professionals could not act on these BPAs. The BPA programming has since been modified so that only physicians are notified. Our evaluation resulted in corrective measures being implemented and serves to highlight the importance of effective pre-launch communication between healthcare teams and BPA programmers as well as post-launch evaluation of EMR BPAs.

Among individuals identified by our BPAs with a positive/inconclusive serology test result, 81% had a subsequent HCV RNA test (before or after a consultation with the TOHVHP). It is unclear why the BPAs were not acted on for the remaining 19% of patients. However, 39% of those with a positive HCV RNA test result identified by the BPAs had a referral sent to the TOHVHP. The influence of the SARS-CoV-2 pandemic on the workload of, and responsiveness to, BPAs is unclear but may have been a factor influencing the impact of our BPAs. To achieve 100% HCV RNA testing and linkage to care, automatic reflex referral submission may be a viable option (17, 24). Linkage to care within a shorter time frame may also be achieved by automated reflex referral.

The population impacted by HCV in Canada is diverse. The prevalence of those testing positive for anti-HCV antibodies is highest among PWID, followed by those with a history of IDU, people who are incarcerated, Indigenous Peoples, and immigrants (7). Our BPAs were designed to identify already tested HCV seropositive individuals and appropriately engage them in care at the TOHVHP, irrespective of risk factor(s). In the majority of previously conducted studies in which EMRs were used to identify and link individuals living with HCV to care, BPAs were configured to recommend screening serology to identify people living with HCV based on either the birth cohort or risk-based screening strategies (13–20). A review of studies found that, by using a risk-based approach for HCV screening, only half of the people living with HCV were ultimately made aware of their status (25). One of the reasons for this suboptimal outcome with risk-based screening strategies was believed to be stigma (25). Furthermore, information related to an individual's risk factors for HCV exposure may be incomplete in an EMR system (17). Considering this finding, we elected to not develop a risk-based BPA screening strategy.

While the BPAs assisted with subsequent testing and linkage to care, many challenges were encountered. From an out-patient perspective, the BPA was configured to be triggered if an individual had a positive serology and/or HCV RNA test result. In our analysis, 39% of consultations to TOHVHP were submitted without confirmatory HCV RNA testing. Of note, reflex HCV RNA testing was implemented in Ontario, Canada, as of April 2023, which has eliminated the issue of consultations being placed without HCV RNA testing and the need for HCV serology-focused BPAs altogether.

The EMR was configured to prompt a BPA for a positive HCV test result within a lookback period of 6 months. If a BPA was not acted upon within the 6-month time frame, then the opportunity to act on this HCV result was lost. The 6-month timeframe limit for HCV testing lookback has been removed so that the BPA will continue to be triggered until appropriate action is taken, and this issue has been corrected. An HCV RNA test posted “Not performed” (due to the absence of serological evidence of past HCV infection) was incorrectly identified as a laboratory result by the BPA algorithm. In our current analysis, 7.9% of the BPA triggers were erroneously triggered by this glitch.

The BPAs were configured differently than intended due to misunderstandings between our healthcare provider and programmer team members. It is essential that instructions are clearly communicated and understood during the BPA construction phase. Staff changes on the BPA programming and clinical teams, as well as competing priorities for programming time, impacted timeliness and team communication. Multiple HCV serology and HCV RNA laboratory test code numbers exist within EPIC to distinguish between onsite and offsite tests. Ensuring that the right laboratory test code numbers were captured before implementation of the BPAs, particularly in the out-patient setting, posed an additional challenge. It is critical that all code numbers are captured to ensure that all patients are identified. The BPA would continue to be triggered until an action was taken and/or completed by a physician. If the BPA was canceled, it would prompt on the screen the next time a patient chart was opened. This prompt provided the responsible physician with multiple opportunities to act on the BPAs. However, this could also lead to scenarios where, if a patient does not return to the hospital, it would result in delays/missed opportunities to engage them in care. BPAs continued to be triggered while patients were actively receiving care at TOHVHP and post-DAA treatment. A “resolved date” is currently being added to HCV patients' EPIC problem lists if started on DAA treatment at TOHVHP or if known to have achieved SVR post-DAA treatment to reduce the triggering of unnecessary BPAs. Finally, an additional burden on physicians to take action as well as frequent BPA prompts can lead to BPA fatigue, influencing physician responsiveness. In our experience, a BPA-related action was taken 15–19% of the time during the period of analysis. We also have determined that a more robust, better-resourced, and persistent education and promotion campaign may have resulted in improved and sustained physician engagement with our BPAs.

Our study findings may not be generalizable or comparable to all settings due to different BPA construction and implementation methodologies. However, we can conclude that our BPA configuration facilitated testing and linkage to care. Furthermore, post-BPA implementation evaluations are critical to assess whether the objectives are achieved and to identify areas for improvement.

## Data availability statement

The raw data supporting the conclusions of this article will be made available by the authors, without undue reservation.

## Ethics statement

Ethical approval was not required for the studies involving humans because this was a cohort research—participants consented to be included in a database that is used for research. The studies were conducted in accordance with the local legislation and institutional requirements.

## Author contributions

ST: Conceptualization, Formal analysis, Methodology, Writing – original draft, Writing – review & editing. RC: Writing – review & editing. NT: Software, Writing – review & editing. JP: Software, Writing – review & editing. RN: Software, Writing – review & editing. GG: Writing – review & editing. CC: Conceptualization, Funding acquisition, Methodology, Supervision, Writing – review & editing.
